# Abstracts &c.

**Published:** 1889-04

**Authors:** 


### ABSTRACTS AND GLEANING.

**Gonorrhœa in the Female.**—A useful article on gonorrhœa in the female is published in the current number of the *Lyon Medical* by M. Horaud. The remarks are based upon 5'090 cases of the disease which had come under the observation of the author. Discussing the microbic origin of the disease, he says that Hallier, of Jena, in 1872, described a micrococcus in the gonorrhœal discharge, and even in the blood of patients who had been attacked with gonorrhœal rheumatism. But the honor belongs to Neisser of discovering, in 1879, the true microbe of gonorrhœa, and of pointing out the method of isolating it and of proving its connection with the causation of the disease. To this microbe Neisser gave the name of gonococcus, which has since been retained. Every discharge from the urethra, vagina, uterus, anus, or the blood of the menses or the lochia, can occasion urethritis in man, but gonorrhœa only follows when the discharge contains gonococci, The purulent ophthalmia of new-born children is only of a gonorrhœal nature when it is contracted from a mother suffering from gonorrhœa, hence it is to be assumed that it is only in certain cases of ophthalmia neonatorum in the pus of which gonococci will be found. The author enters fully into the subject of how to collect pus for examination for the gonococcus from the female generative organs. Urethral gonorrhœa may persist a long time in women—some months, and even years. He has seen patients the pus from whose urethra still contained gonococci at the end of four and ten months, and of two years, without reinfection having taken place to account for the disease. No cases of auricular blenorrhagia, nor of nasal nor buccal, had come under his observation. A short time ago, however, he published a note of urethral gonorrhœa in a man which had been contracted during a coitus, *ab ore*. The woman from whom the contamination came, and who had lent herself freely to this kind of occupation, submitted to a minute examination, but no buccal lesion was detected, nor were there any traces elsewhere about her of gonorrhœa. The author concluded that the contagion had been derived from the gonococci deposited in the mouth during a previous coitus. In the treatment of gonorrhœal discharges he speaks highly of nitrate of silver, both in solution and in the solid form. Even weak solutions of the drug of 0.30 of a gramme to 100 grammes of distilled water are successful in reducing old chronic discharges. From comparative trials which he has made of nitrate of silver and of sublimate solutions, both in cases of urethral gonorrhœa and purulent ophthalmia of infants, he is confident that the former drug is in every respect superior, and yields better results.—*Medical Press and Circular*.

**Ventro Fixation in Retroflexion.**—At the Societe de Chirurgie M. Terrier spoke on "ventro-fixation" for retroflexion of the uterus. In March last he operated on a woman for a very painful and obstinate displacement. Digital examination revealed a small tumor on the left side, very sensitive to the touch, so much so that he first thought of salpingitis. However, he was soon convinced that the case was one of obstinate retroflexion, and that the tumor was one of the ovaries. Laparotomy was performed without delay, the body of the uterus fixed to the abdominal wall by two sutures, the ovary reduced, and the patient made a rapid recovery, A second patient was handed over to him by one of his colleagues of the hospital for a painful retroversion. Examination was very difficult, from tenderness of the parts. A similar operation was promptly performed, and the uterus fixed by means of four sutures. M. Terrier concluded by saying that he claimed for himself the honor of being the first to perform this operation in France. M. Despres criticised this new method of treating displacements of the uterus. He considered that rest with a hypogastric belt was sufficient to bring about a cure, and characterized the operation as audacious surgery which should not be encouraged. M. Lucas said he had operated twice for the affection in question, and he could not help feeling astonished at the result. He could not entertain the fears of M. Despres, and promised himself to operate in every other case presenting the same difficulties. This method is better than Alexander's in that where the appendices are diseased they may be removed at the same *seance.*—*Med. Press and Circular.*

**Antipyrin and its Substitutes.**—Numerous enquiries have becn addressed to us on the question of substitutes for antipyrin. Medieal journals in the country have published reports of the French substitution, called Analgesine, and the Chicago *Medical Standard* adds to the confusion by urging the adoption of the name Methozin in place of antipyrin.

The simple letter of the U. S. patent law prevents the sale in this country of antipyrin under any other name, or of any other manufacture than that of Meister Lucious & Bruning (Dr. Knorr), because the substance itself and the process of manufacture are patented. It is idle, therefore, to speculate on substitutes; but an excellent opportunity presents itself thereby to bring an arbitrary, impolitic and extremely foolish patent law into general disrepute, and it may help to finally amend the same.

It may be added that the French government protects its own subjects differently. In France the name Antipyrin is protected and the sole property of its original owners but the chemical can be made and sold under any other name by any manufacturer. This is analogous to the position occupied by antifebrin, the copyrighted name for the chemical acetanilin.

It is also permissible in this country for physicians and druggists to arrange mutually to prescribe and dispense antipyrin under the *Medical Standard's* name, Methozin. This would serve no logical purpose, however, and might—if widely practiced—lead to legal proceedings.

Inasmuch as our patent laws give such arbitrary protection to alien inventors, who cannot even enjoy approachable privileges in their native countries, we should yield to circumstances and act within the restrictions of these laws. And if any of us have public influence let us work tooth and nail to revise our patent laws. Revision of tariff and of patent laws go well together, as both tend to create dangerous and unjust monopolies that should have no existence.—*Notes on New Remedies*, by Lehn & Fink.

**Papein**, the vegetable digestive ferment obtained from the unripe fruit of *Carica papaya*, or Papaw, has been again brought into notice as an active alternate to pepsin. While pepsin is inactive in neutral and alkaline conditions, papain is active, and therefore, in using the latter it is not necessary that any free acid should be present. Pepsin with acid, and papain without, are claimed to be equal in activity and power, and if so, there are quite a number of conditions to which papain is applicable to which pepsin is not. It, therefore, appears rational that in dyspepsias, which are not relieved by pepsin, papain may, as is asserted, be useful, and thus become an additional resource for the practitioner."—Dr. Squibb's *Ephemeris.*

**Dyspeptic Dyspnœa.**—*N. Y. Med. Jour.*, December :—Dyspeptic dyspnœa is characterized by a sense of weight or oppression across the chest, an almost constant desire to draw a long breath, and a feeling that the air does not enter the lungs to a sufficient depth. Repeated inspiratory efforts are made. The body is fixed in some certain position or a sudden quick change of position is made before a satisfactory inspiration is obtained, but the relief is of short duration, and these efforts must soon be repeated.

Diversion, intense occupation, or active bodily exercise, such as rapid walking, sometimes mitigates or relieves the attack. None of these ever aggravates it. Dyspeptic dyspnœa occurs after meals, in the intervals of stomach digestion, and not infrequently at night. The duration of the attacks is variable, but they usually extend over a considerable portion of the day or night. They recur daily, or at intervals of a few days, for weeks or months, or there may be but a single attack.

There is a more alarming form of dyspeptic dyspnœa, the paroxysms of which occur at night only. Attention was called to this a few years ago by patients who presented themselves complaining of severe attacks of shortness of breath or smothering coming on at night, and unaccompanied by wheezing, as in asthma.

Physical examination of the chests of the patients gave no signs of pulmonary or cardiac disease, and the urine gave no evidence of kidney disorder. The only accompanying symptoms that could be obtained related to the digestive organs. This variety is less frequent than the milder form of dyspnœa previously described. The writer has seen only a few examples. The paroxysm occurs usually after the patient has gone to bed. He is awakened out of sleep with a feeling of weight across the chest, a distressing sense of suffocation, and inability to get his breath. He sits up quickly in bed and makes strong efforts to inspire, but the air does not seem to enter his lungs. He becomes nervous and anxious. No position gives comfort. He gets out of bed, sits in a chair, walks the floor, probably goes to an open window, lies down, sits up again, but obtains no relief. Becoming excited, his heart beats rapidly, and this increases his distress and anxiety. The respiration is slow, irregular and sighing. The expression is one of great suffering. The face is pale—the surface is cool. Perspiration may appear on the forehead only, or involve the whole body. Examination of the lungs reveals no abnormal signs except possibly feebleness of the respiratory murmur. The heart's action is usually rapid and irregular; it may be slow and regular. No albumen is found in the urine, but this secretion is sometimes dark and of high specific gravity, depositing urates and uric acid.

The attack lasts from half an hour to an hour or more. It subsides gradually, and the patient falls asleep; but he may be awakened by another before morning. The attack may recur several successive nights, or at longer intervals over a considerable period. There may be but a single attack.—*Epitome.*

**A case of Sciatica treated with large doses of Antifebrin.** —By Austin Flint, M. D.—*N. Y. Med. Record*, December 18.—M. G——, aged twenty-five, laborer, was admitted to ward 19, Bellevue Hospital, August 14, 1888. The family history was negative. As regards the previous history there is nothing special to note until about a year ago, when the patient had pain, with soreness and tenderness at the back of the right thigh, extending as far as the knee. This became so severe that he was compelled to keep the bed for two or three weeks. Under treatment at that time he improved, but has not been entirely free from pain since.

For the past three weeks the pain has been so severe that the patient has been confined to the bed most of the time. He has been treated with blisters, iodine, and a variety of internal remedies. Electricity had been tried before his admission to the hospital, but this gave no relief.

August 16, 1888.—The right leg was buried in flowers of sulphur for thirty-six hours. The patient said the pain was "perhaps a little better" after this application.

August 18.—Sulphur was again applied for forty-eight hours but without relief. At the end of that time the urine had a distinct odor of hydrogen monosulphide.

August 25.—The patient was brought under influence of ether, and the nerve was stretched by forced flexion of the thigh. This gave no relief.

August 26.—I requested Dr. Rutsen Mauray, the house-physician, to administer antifebrin in as large doses as could be borne, watching the patient carefully. This treatment was carried out even more vigorously than I had expected. At 11 A. M. Dr. Mauray gave twenty grains of antifebrin; at 1 P. M. fifteen grains, and at 3 P. M. fifteen grains, making in all fifty grains within four hours. The patient became somewhat cyanotic, and a half-ounce of whiskey was given with the last dose.

August 27.—The pain was nearly, but not entirely, relieved and the antifebrin was given again in the following doses: 10 A. M., twenty grains; 12 M., twenty grains, making forty grains within two hours. It was not necessary to give whiskey.

August 28.—The pain was completely relieved, and the patient walked about the ward without difficulty. The large doses of antifebrin taken on August 26 and 27 had no unpleasant effects, and the patient expressed himself as feeling perfectly well, absolutely free from pain, and able to move about as he did a year ago, before he was first attacked.

August 31.—The patient was discharged. He insisted on leaving the hospital to go to work. He promised, however, to report himself in case the pain should return. At the date of writing (November 12, 1888), no report from the patient has been received.—*Epitome.*

**Pleurodynia.**—Tinct. iodine painted over the chest, will often relieve pleurodynic pains where mustard plasters fail.

**Pneumonia.**—Dr. Barnes, in *Med. Brief* says:—My treatment for pneumonia is to give the fluid extracts of veratrum viride and aconite, two drops of each, every two hours, until the circulation is perfectly controlled, then at longer intervals. Commencing early in the disease.

**On the administration of pichi.**—Dr. C. E. Nash, Little Rock, Ark., sends the following notes on the administration of pichi:

This remedy, after an employment of only a few years, has gained an enviable reputation in the treatment of a number of urinary affections, in nephritis, cystitis, acute gonorrhœa, vesical irritation, etc. Its habitat is South America, and since its introduction it has gained many enthusiastic admirers both here and in Europe. An examination of its constituents indicates that the activity of the drug may be referred principally to a bitter resin, which is not abundant, a volatile oil and perhaps also to two other principles which however exist in only moderate quantities, viz: æsculin and a body having the properties of an alkaloid. Its action is that of an urinary diuretic and sedative, besides exhibiting also undoubted antiseptic properties, these latter being shared by other oleo-resins.

Owing to the large amount of resinous matter in a necessarily alcoholic extract, if the latter be mixed with aqueous liquids, a considerable quantity of the resin will separate in clots which would of course impair the efficiency of the remedy.

In searching for a suitable vehicle the writer finds that mucilaginous or syrupy liquids should have the preference and if possible avoiding saline ingredients as these have a well known disposition to separate resins, in like manner as they do soaps, from solution in form of a curd. The most eligible vehicle however for the fluid extract is glycerine which appears to be a fairly good solvent as when the mixture is diluted with water prior to administration, the sparingly soluble resins are suspended in a state of extremely fine division.

Many inquiries have been made for a form in which this remedy could be used hypodermically but the difficulties in the way are apparently insurmountable, owing, 1st: to the sparing solubiliity of the active ingredients in aqueous fluids or such as are suited for injection, and, 2nd: The amount of the dose.

A most important feature should here be borne in mind, and that is, the effects of the remedy must to a large extent be considered local, just as in the case of cubebs or copaiba, and that the hypodermic method would certainly be an extremely roundabout way of bringing the remedy into contact with the surfaces affected. It could reach them at best in a very attenuated form. Should any of our readers have the courage to try it in hypodermic form, it should be remembered that glycerine if used as a solvent, is itself irritating unless entirely pure and slightly diluted with water. A solution for trial should also be carefully filtered through absorbent cotton after standing at least 24 hours, to separate any suspended waxy matters.

For convenience of administration, Messrs. Parke, Davis & Co., have recently issued a new preparation of this drug which appears quite eligible, namely a powdered extract. Owing to the readiness with which this can be given in capsule (a very desirable form for administration) pills, or even suspended in water or milk the taste may be, to a considerable degree obviated. The dose of the powdered extract is from five to ten grains and as the active principles are here presented in a minutely divided form, exposing a large surface for solution to the fluids which largely find an exit through the renal tract, it would naturally commend itself as an improvement worthy of trial.—*Med. Age.*

**Treatment of Erysipelas.**—Dr. Nolte (Allgem. med Centr. Ztg., No. 100, 1888) has in the treatment of this affection for years used mucilage of gum arabic with carbolic acid. The affected parts are painted daily with mucilage containing from a three to five per cent. carbolic acid solution, and allowed to dry.—*Ex.*

**Treatment of Deafness of Old Age.**—Sapolini, of Milan (International Congress of Otology, Brussels, September 10-14, 1888), describes a method of his, which he states he has successfully employed in sixty-two cases of deafness of old age. It consists in mopping the membrane tympani with a weak oleaginous solution of phosphorus. He claims that this treatment diminishes the opacity of the membrane, increases the circulation, and improves the hearing.—*Amer. Jour. of Med. Sciences*, Jan., '89.

**Graves' Disease.**—*Sulphate of Spartein in.*—The effects of spartein in three cases of Graves' disease were remarkable, and if a wider experience confirms them, it will be a valuable remedy in this disease. In two of the cases the usual remedies, including a long course of arsenic, and the passing of a constant current through the neck, etc., has been tried without effect. On giving spartein the pulse rate dropped in one week from constant rates of 132 and 136 to the minute, to rates of 72 and 84 respectively, the coincident throbbing and pulsation of the thyroid and of the great vessels of the neck ceased. Other symptoms were relieved, especially the nervousness and weakness previously complained of, and the size of the thyroid tumor distinctly diminished. The gain in strength and general well-being, with improvement of appearance due to gain of flesh and color, was striking. In the third case, a more severe example of the disease than either of the preceding, there was general improvement, with diminution of violent pulsation in the thyroid and over the neck and face, but the pulse-rate of 144 was not reduced to less than 96 to the minute, varying between this and 108. This patient suffered from continual fine tremor of the muscles, especially in the limbs; while taking spartein this tremor remained absent, but returned if the drug was left off. She has improved in strength and feels well while she is taking it. She has now been taking one-quarter of a grain every four hours for six months.—*Dr. J. Mitchell Clarke, p.* 171.

**Scarlantina and Bronchitis among Children.**—*Antipyrin in.* —Friedlander, of Russia, reports fourteen cases of scarlantina, all of which recovered under the use of antipyrin in doses of nine grains once to three times in twenty-four hours. Profuse perspiration, fall of temperature, and in nearly all cases refreshing sleep of two hours, followed the use of the drug. The dosage was according to age, the maximum dose is that stated. In acute bronchitis nine grains of antipyrin sufficed to influence children under two years of age favorably for twenty hours; while thirteen grains, with children under five years of age, were sufficient to lower temperature for twelve or fifteen hours; this dose was generally given twice in twenty-four hours. Profuse perspiration, lessened cough, sleep, and general improvement followed. During convalescence, half the doses given during the height of the disease were used. In addition, the following was found useful: ℞. Caffein, gr. $\frac{9}{10}$ to gr. 3; sodii bicarb. gr. 23 to gr. 45; aquæ fœniculi ℥15; syr. ipecac. ℥7½. M. Sig. One-half to one teaspoonful every half hour or every two hours.—*Medical News, Oct. 8, p.* 423.

**Brief outline of the Teachings of Surgery in France.**—Many recent American graduates do not judge their education complete without a more or less prolonged stay in Europe. We take this occasion to say a few words regarding the methods of teaching surgery in France:

France of late years has been little considered as a medical centre. It is true that there is at present no shining galaxy of surgical lights as thirty years ago when Nelaton, Velpeau, Maissoneuve and Chassagnac held their clinics. But there are a number of younger surgeons not of world-wide fame, but who nevertheless do excellent work. Among these may be mentioned Verneuil, Le Dentu, Lucas-Champiouniere, Terrier, Perrier and Trelat, as gynæcologists Pollailon, Terrillon, Pozzi and Apostoli.

The name of Ollier of Lyons is doubtless familiar to all readers of bone surgery; unfortunately his clinic is scarcely visited by foreigners. It is to Ollier that we owe many of the recent advances in operative bone surgery, and it will fully repay any young graduate to spend a few weeks in Lyons, where he will be sure of a cordial reception. In the wards of the Hotel Dieu of that city he will be able to study M. Ollier's methods of operation, many of which have not yet been described in the text books. His museum though small, is one of the most interesting in the world, as it contains specimens of cases operated on by him, and the results as gained from autopsies many years after the operation.

For the study of anatomy, Paris is the place *par excellence.* This branch of medicine can be studied in two ways: either by matriculating and dissecting at the Ecole Pratique, and listening to the lectures of Professor Poirrier and his assistants, or still better by going to Clamard in Rue du Fer a Moulin and paying ten dollars a month, where for that price private instruction and all desirable material can be obtained. In the latter institution private instruction in operative surgery is also given, which is unexcelled on account of the large quantity of material and the small number of men in each class.

Antisepsis is thoroughly carried out by the younger surgeons, and the antiseptic methods of the French must not be judged by seeing Pean and Le Fort operate. The French surgeon is an excellent diagnostician, although not as good a pathologist as his German confrere, but he has the advantage of being more cosmopolitan in his views, adopting whatever seems best to him from every country. French surgery closely resembles that which we see practiced every day at home, being extremely conservative, but at the same time sufficiently bold when the occasion demands it.—*Int. Jour. of Surgery.*

**Magisterium Bismuthi in Infantile Summer Diarrhœa.**—In the St. Petersburg weekly *Ruskaia Meditzina*, Dr. A. Puginoff emphatically declares that subnitrate of bismuth constitutes the most reliable remedy for epidemic summer diarrhœa iu nurslings. He gives the drug *larga manu*, feeling sure that a pure preparation is excreted *per anum* wholly and in an unaltered state. Thus, to an infant 4½ months, he administers 1½ or 2 grains every 2 hours. The main advantages of the subnitrate over all other means are stated to be these: 1. The drug does not give rise to any untoward accessory symptoms. 2. It is readily taken and perfectly well borne. 3. It acts on the intestinal tract both as a sedative and as an antiseptic.—*St. Louis M. Journal.*

**To Blister the Skin Quickly.**—The following methods are rapid in their action, and although not new they will bear repetition. The *Medical Record* gives them in brief as follows: Into a watch glass, pill box or any similar receptacle, pour ten drops of concentrated water of ammonia (aqua immonia fortior); cover the liquid with a little bit of linen or cotton wool, and at once apply the cup upon the skin where the blister is required. Press so that the vapor is confined to the inside of the vessel. A red circle will directly be observed outside, when it will be certain vesication has taken place. Half a minute or so is all the time required to obtain the result. The blister may be dressed in the usual manner of dealing with a blister from cantharides. Concentrated acetic acid applied to the skin, will also in a few minutes produce vesication. In such cases evaporation should be prevented by some suitable covering. Bibulous paper slightly wetted with a little of the ethereal extract of cantharides instantly applied to the skin and covered with a piece of adhesive plaster will answer for the same purpose.—*Ib.*

Thomas Oliver, M. D. (*Lancet*, Nov. 24), says of Adonidine: The cases in which I have tried it have been chiefly those of aortic and mitral regurgitation. In all of them great relief was given to præcordial pain, to the pain which ran down the left arm, and to palpitation and dyspnœa. I give it, combined with such things as sal volatile or chloroform water, in doses of one-sixth of a grain four times a day. It is a powerful drug, and is not borne well, particularly at first, in doses larger than this. Frequently, in fact, I begin with one-eighth of a grain, and I find this quite enough. Every patient has expressed himself after a few days of trial of it as feeling very much better, and not in one direction only. The painful throbbing of blood vessels, headache and profuse perspirations have in addition to dyspnœa and præcordial pain, disappeared; and whilst in most of the cases there has been a slight increase in the amount of urine thrown out after treatment, I am inclined to think that adonidine has little diuretic action. It is a cardiac tonic.—*Medical Chips.*

For the gastro-intestinal catarrh of drunkards, two or three drop doses of Fowler's solution before meals is a sovereign remedy.—*Ib.*

**Boiled water.**—The *Gazette de Gynakol.* urges the use of boiled water as an antiseptic, which after filtration is applied to wounds at a temperature of 100 to 120 degrees, is the best antiseptic in use. [The late Dr. Varick of Jersey City, demonstrated to the satisfaction of all surgeons that water at this temperature is as good, if not better than any antiseptic solution. The experiences in the St. Louis City Hospital have supported this view. May not the good results of water at this temperature be due to a slight coagulation of albumen?]—*Ib.*

**Epistaxis from Salicylism.**—That the salicylates produce a hemorrhagic tendency is now conceded. A writer in the *Lancet* remarks: That they do cause epistaxis is established beyond all doubt. The occurrence is mentioned by nearly all writers on this subject and the fact was freely accepted by the physicians who took part in the discussion at the Clinical Society in 1881. Of 174 patients treated at Guy's Hospital by these drugs, I found that 6 per cent were recorded to have had this symptom. Thus the difficulty is not to establish the hæmorrhage but to satisfactorily explain how it is produced. In the first cases which I observed, I was struck by the bounding pulse which preceded the bleeding, and thought that some increase of arterial tension might be an important agent, but a more careful investigation showed that although the pulse was large and forcible, it was of decidedly low tension, a fact which was confirmed by sphygmograms taken before, during, and after the period when the patient was thoroughly under the influence of the drug. It seems, therefore, that we must fall back upon some chemical or physical change in the blood which makes it more readily transude through the capillaries, or else upon some secondary change in the walls of the vessels themselves. The point of practical importance to which I would call attention is this: Although I have carefully watched these cases for some years, I have never observed epistaxis or any hæmorrhage occur until several hours, and generally not until several days after the more common symptoms produced by too large a dose have been well marked. These symptoms are deafness, headache, vomiting, tinnitus aurium, and an irregular and slow pulse, this being the order of frequency with which they occurred in the series of 174 cases, before referred to. It would, therefore, seem if due regard were paid to these indications that the drug is beginning to produce its physiological effects, and an appropriate alteration made to the dose, the occurrence of loss of blood which the patient can so ill afford, might be prevented.

**Antipyrin in Laryngismus Stridulus.**—Mr. Montague Perceval (*Lancet*) states that he has recently treated twenty-four cases of laryngismus stridulus with antipyrin, administering two grains every hour. In these cases with one exception these paroxysms were arrested; the case where this did not occur, increasing the dose to five grains also served to relieve the dyspnœa. He has used this remedy successfully in cases where an emetic dose of ipecac had failed.

**Dangerous Antipyrin Compounds.**—Antipyrin when combined with spirits of nitrous ether containing any free nitrous acid, produces a poisonous compound, which may be called nitroso-antipyrin. It is characterized by a greenish color after standing, and care should be taken by physicians not to prescribe them together. The chemical action which occurs is expressed as follows:

$$C_{11}H_{12}N_2O + HNO_3 = C_{11}H_{11}N_3O_2 + H_2O.$$

Antipyrin + Nitrous Acid = Nitroso-Antipyrin + Water.

From experiments it is also inferred that antipyrin with belladonna makes a dangerous eompound, the patients suffering sevcrely from respiratory oppression, cerebral pressure, sensation of great discomfort and fright. Much care should be exercised in prescribing antipyrin in combination with other remedies until it is better known.—*The Formulary.*

Sir Morrell Mackenzie lately visited the Edinburg Eye, Ear and Throat Hospital, and, in the course of a short clinical lecture on Acute and Chronic Tonsilitis, expressed his preference for guiacum as a remedy at the outset. He has the guiacum made into lozenges containing about three grains each. Nine cases out of ten will rapidly get well, if one of these lozenges is given every two hours at the commencement of the attack of tonsilitis. He sometimes also applies locally, a little bismuth and opium, or an eighth of a grain of starch, because the problem is not only to cure the patient, but to keep him comfortable till he is cured.

Sometimes the guiacum causes some diarrhœa, but this is rather an advantage, and the morphia usually soon checks it, as well as allays the slight stinging sensation produced by the guiacum upon the throat. He uses this treatment in acute inflammations of any part of the back of the throat, and after an experience of fully twenty years pronounces it really specific.—*Ib.*

In a communication to the *Md. Med. Jour.*, Dr. Edward Anderson, of Rockville, recommended the use of the Bitart. Potass. in the latter stage of gestation, as a preventive of Puerperal Eclampsia. This has been endorsed by the Gyn. and Obst. Soc., of Baltimore, and now the Doctor adds, that the bitartrate will not only prevent convulsions during pregnancy, but will also prevent their occurrence in Bright's disease, and in albuminaria following scarlet fever. The potass. is to be given at once when a trace of albumen is detected in the urine.

**The Antilithic Properties of Antipyrin.**—In the *Revue Generale de Clinique et de Therapeutique* for Januaro 24, 1889, H. Huchard writes that about a year ago he administered several doses daily of 15 grains of antipyrin for violent renal colic. The result in relieving pain was very striking, so much so that the patient contracted the habit, without advice, of taking two doses of 15 grains each of antipyrin every day. He continued this independent treatment for about six months, and then reported that his pain had entirely disappeared and that there had also been a great change in the renal Secretion. The urine was entirely free from sediment, was clear, limpid, and chemical examination showed that it contained but a normal quantity of uric acid. This result was especially surprising, since it is generally acknowledged that antipyrin diminishes renal secretion. Dr. Huchard states that he hardly felt warranted in concluding that the improvement in this case was attributable to the antipyrin until he found a case reported by Schweig which had likewise been relieved not only of the renal colic, but that the renal secretion had been restored to its normal character by the daily administration of 1½ grains three times daily. These two observations are naturally not sufficient to establish the remedial value of antipyrin in the uric acid diathesis, though it seems to warrant further experiments being made with this drug in this connection.

**Juniper Berries as a Diuretic.**—According to the Paris correspondent of the *British Medical Journal* for January 12, 1889, Dr. Goldschmid, of Fehraltorf, highly praises the inspissated recent juice of common juniper berries as the best diuretic in children. Attention was drawn to this remedy by Professor J. Vogel, of Dorpat, in his classical handbook on children's diseases. While being most effective, the remedy is exceedingly mild and altogether free from any unpleasant accessory effect. Two or three teaspoonfuls should be given daily, diluted with water and sweetened with sugar. The little patients take it very readily. The author describes a severe case of nephritic dropsy in a girl, aged 7, where the juice rapidly induced a profuse diuresis, a complete and permanent recovery ensuing in a fortnight.—*Ib.*

**Antipyrin in Obstetric Practice.**—In the *Northwestern Lancet* for January 15, 1889, Dr. Talbot Jones reports seven cases of labor in which rectal injections of about 30 grains of antipyrin were employed to relieve the suffering from labor-pains. With one exception, he claims that the pain almost wholly ceased, although the uterine contractions continued without alteration or diminution until the birth of the child. He likewise claims that it will produce great relief in obstructive dysmenorrhœa and the after-pains of child-birth. It is to be noted that this is not in accord with the experience of Dr. Huchard as detailed in this issue of the *Gazette.*

**Chorea.**—*Antipyrin.*—In a communication that recently appeared (*Revue de Therapeutipue*) M. Legroux give the results obtained from the use of antipyrin in chorea. By the treatment hitherto pursued, the average duration of the cases of chorea has been sixty days according to M. M. See and Rogers, and ninety days according to Cadet de Gassicourt, but in the treatment by antipyrin Legroux finds that the average duration is about sixteen days, the minimum being six, and the maximum twenty-seven days. The mode of administering antipyrin is simple. In twenty grammes (about six drachms) of syrup of bitter orange (French codex) he dissolves one gramme (fifteen and a half grains) of purified antipyrin. Three doses of this size are given in the course of twenty-four hours.—*American Journal of Med. Sciences*, *March*, *p.* 283.

**Electric Illumination of the Male Bladder and Urethra.**—At the Medical Society of London, on January 23, Mr. Hurry Fenwick showed a series of instruments made by Leiter, of Viena, and Hartwig, of Berlin armed with incandescent lamps for illuminating the male bladder and urethra, and demonstrated their capabilities upon patients and "dummies." He observed that endoscopy had attracted the attention and efforts of the profession since the commencement of the century, but the candle power hitherto used had proved insufficient. Quite recently (1887) however, the smallest Edison lamp had been employed, and the result had been brilliant. Instead of a cumbersome, costly, and fickle instrument like the Nitze-Leiter endoscope of 1880, we now possessed a simple, practical, and safe apparatus by which the bladder or urethra could be examined in as strong a light as if it was viewed by direct sun-light. (See woodcut and description at p. 160 of this volume.) Every detail of the vesical or urethral surface was discernible, under favorable circumstances, even to the small vessels coursing over the mucous membrane. Mr. Fenwick mentioned certain elements necessary for a successful bladder examination, and explained that the vesical endoscope needed much practice and patience before the observer, could become proficient with it. He believed that the value of electric endoscopy could hardly be estimated, and he predicted that it would at once assume a high rank in the diagnosis of obscure vesico-urethral disease, and would become almost as indispensable as the opthalmoscope or laryngoscope. (Mr. Schall showed various batteries for working the endoscopes.) Mr. Walsham bore testimony to the ease with which he had been enabled to see a stone in a dummy bladder.—*British Med. Jour.*

**Coryza.**—*A snuff for.*—M. Pierre Vigier considers a powder composed of equal parts of powdered starch, boric acid, and tincture of benzoin to possess certain advantages, especially that of not being too light. The mixture should be triturated for a moment, then dried with a gentle heat, and put into a box, without pushing the powdering process too far. The rapidity of the effect is proportionate to the amount of the powder used and the frequency with which it is employed.—*New York Med. Journal.*

**Dangers of Sulphonal.**—Sulphonal, the now fashionable hypnotic, is the subject of very varied professional opinion. Some extol it, others condemn it. The truth probably lies, as usually happens, between the extreme statements. Sulphonal has a clearly defined usefulness, and belongs not so much to the class of narcotic agents which produce sleep by stupefaction as to the remedies which assist the natural periodical desire for sleep. The drug is, however, by no means so harmless as has been hitherto asserted by its manufacturers. Dr. Bornemann has just reported a severe case of poisoning resulting from the administration of the drug. The patient to whom sulphonal was given for insomnia caused by cerebral excitement was a physician. The result was a pronounced intoxication showing very complicated symptoms. There was a distinct interference of coordination, first in the lower, and later in the upper extremeties. He could not, for instance, raise a cup of coffee. A very prominent feature of the poisoning was his perverted feelings and illusions. The patient believed he had two heads, four feet and arms, etc., or believed he was on a boat or in a railway car, and that some one was about to kill him. These illusions may be termed reflectory. The ataxia referred to is a central one, as it remained unchanged no matter whether the eyes were opened or closed. This distinction between central and sensory ataxia has been made by Prof. Mendel. The drug did not exert any unfavorable influence over the heart and circulation, which appears opposed to the warning of Dr. Schmey not to use sulphonal in angina pectoris and arterio-sclerosis.—*Med. and Surg. Reporter.*

Dr. A. H. Newth (*London Lancet*) recommends the hyposulphites in the treatment of the bites of rabid dogs, and also their administration after the development of hydrophobia.

After a bite by a mad dog, he would give (to a child) five or ten grains of the hyposulphite of sodium or magnesium (the latter is richer in sulphurus acid), in caraway water with sirup, every four hours for the first three or four days, then three times a day for a week, then every morning early for one month; recommending a Turkish bath twice a week. When the disease has developed, he prescribes the hyposulphite every hour, with hot air baths to induce perspiration. The hypodermic injection of hyposulphite might also be tried, especially if the patient is unable to swallow. Dr. Newth has used this remedy repeatedly in cases of blood-poisoning, with the most marked success; as, for example, a patient has received a punctured wound which has inflamed, the lymphatics have become swollen and reddened, the parts are extremely painful, and there are rigors. Within a short time after the exhibition of the hyposulphites, the pain has decreased, the parts are less inflamed, and symptoms of poisoning have abated. Nearly thirty years ago Professor Polli of Milan suggested the use of sulphurous acid in cases of blood poisoning, and proved by experiments that dogs that had putrid blood injected into their veins, quickly died; if the hyposulphite of sodium was previously mixed with the injected blood, they were not affected; also if the hyposulphite was administered to the dogs either before or immediately after the injection of putrid blood, they did not suffer therefrom.—*Ib.*

**Monthly Summary of Medical Progress.**—Dr. Schmidt (*Berlin Klin. Woch.*) in an article upon diseases of the Antrum of Highmore, describes a simple method for diagnosing collections of pus or other fluids in this cavity. Shut in by bony walls, catarrhal or purulent inflammations of the mucous lining of the antrum may continue a long time, causing only obscure pains in adjacent parts, which may be attributed to neuralgia.

When the natural opening into the nasal cavity is closed, and when the patient objects to losing a tooth, or has no upper tooth on that side to lose, the method of Dr. Schmidt is advisable.

It consists in placing a small pledget of cotton, saturated with a 20 per cent. solution of cocaine, just below the antrum end of the lower turbinated bone, and leaving it in that situation for ten minutes, after which, with a very strong syringe-needle, the anterior end of the turbinated bone is raised and the needle is driven diagonally outward in the direction of the external opening of the ear. If the first attempt fails, another should be made a little higher and further back.

In most cases the wall of the antrum may be pierced at one or other point, and pus may be drawn into the syringe. Careful disinfection is enjoined. The syringe-needle used in this operation should be twice as long and more than twice as thick as the ordinary hypodermic needle, with a curve like an ear catheter. Its point should be rounded, with a sharp edge. The operation is almost painless, and has no disagreeable consequences; but for repeated irrigations and prolonged drainage, a large opening through the alveolar ridge is better than a large one through the walls of the nasal cavity.—*Popular Science News.*

In *La Progres Medical*, Professor Adomkiewicz claims to have obtained remarkable results from the combined action of chloroform and the constant electric current, in facial and other forms of neuralgia. The electrode is made of hollow charcoal, into which the chloroform is introduced, and from which the current sends it into the tissues.

That this power of penetration may be thus obtained, is thought to be shown in the fact that when chloroform is colored with gentian violet, and applied as described to the ear of a rabbit, the tissue becomes dyed.

In the human subject, the action of the constant current and the chloroform produced a burning sensation, followed by anæsthesia, except where the nerves are deep-seated, as in sciatica.—*Pop. Science News.*

